# The correlation between pregnancy-related low back pain and physical fitness evaluated by an index system of maternal physical fitness test

**DOI:** 10.1371/journal.pone.0294781

**Published:** 2023-12-21

**Authors:** Longfeng Zhou, Xiaoyi Feng, Ruimin Zheng, Yuhan Wang, Mengyun Sun, Yan Liu

**Affiliations:** 1 Institute of Physical Education and Training, Capital University of Physical Education and Sports, Beijing, China; 2 National Center for Women and Children’s Health, Chinese Center for Disease Control and Prevention, Beijing, China; 3 Department of Pharmacology, Addiction Science, and Toxicology, College of Medicine, University of Tennessee Health Science Center, Memphis, Tennessee, United States of America; UFPE: Universidade Federal de Pernambuco, BRAZIL

## Abstract

To investigate incidence of pregnancy-related low back pain (LBP), evaluate physical fitness objectively during pregnancy and analyze the correlation between LBP and physical fitness of pregnant women, 180 pregnant women including 101 in mid-gestation (14–28 gestational weeks) and 79 in late-gestation (28–37 gestational weeks) were recruited and self-reported their LBP. The aerobic ability such as cardiorespiratory fitness and anaerobic ability including strength, endurance, speed, flexibility, and balance were evaluated by a novel materal physical fitness test system. The correlation between LBP and each component in physical fitness test system was analyzed in SPSS. As the results, 135 out of 180 participants (75% of total) had pregnancy-related LBP. Physical fitness of participants in late-gestation was significantly weaker including weaker back strength (p<0.05), less resistance band pullbacks in 30s (p<0.01), less stretching in sit-and-reach test (p<0.001), shorter duration in left legged blind balance test (p<0.05) and weaker bird dog balance(p<0.05) than those in mid-gestation. Correlation analysis indicated that LBP was negatively associated with standing heel raises in 20s (p<0.01) and standing glute kickbacks in 30s (left p<0.01, right p<0.05). Thus, it is concluded that LBP is in high prevalence throughout the entire pregnant course. The pregnant women are prone to have weakened strength of core muscle groups and poorer flexibility and balance along the pregnancy. In addition, their LBP was negatively correlated to strength of back muscle groups of lower limbs.

## 1 Introduction

Pregnancy-related symptoms are experienced among a majority of pregnant women and may reduce the quality of life [1, 2] and adversely affect mental health, birth outcome and postpartum recovery [3–5]. Low back pain is one of the most common symptoms in pregnancy [6]. Statistics suggest that more than 50% of women experience low back pain during pregnancy [7], and more than 30% of pregnant women will continue to be affected in the postpartum period [8]. Considering the safety of fetal development, pregnant women prefer conservative treatments such as support belt use, kinesio taping, activity reduction and even bed rest to relieve low back pain [9–11]. However, use of different support belts [12] and bed rest [13] are proved to have no significant effect on low back pain and the efficacy and safety of kinesio taping for pregnant women is still in debate [11]. Actually, exercise and physical therapy are the first-line treatments as non-pharmacological methods regarding to general low back pain [14]. Increasing evidence shows that low back pain such as sacroiliac joint mechanical pain and herniated discradicular pain are accompanied by spine tenderness and muscular weakness [14]. To treat these types of low back pain, improving physical fitness such as movement control exercise designed to strenghthen muscles and improve spinal posture has a positive effect on disability as well as pain alleviation [15].

With growing understanding of safety of exercise during pregnancy, physical activity during pregnancy has been a common sense throughout the world [16–18]. All the pregnant women without contraindications are strongly recommended to have certain exercise to avoid pregnancy-related symptoms according to the WHO guidelines (2020) and other reports [19–21]. In recent years, more and more exercise prescriptions have been designed to treat pregnancy-related low back pain [22–24]. However, a review covering 34 trials examining 5121 pregnant women provided low-quality evidence that any land-based exercise significantly reduced pain [12]. This low-quality evidence and diversity of exercise prescription used bring us back to reconsider the relationship between pregnancy-related low back pain and individual physical fitness.

It is reported that greater self-reported overall physical fitness assessed by International Fitness Scale (IFIS) was associated with pregnancy-related lumbar pain [25]. Meanwhile daily activities based on free descriptive answers including standing up from chair, lying down, tossing and turning were found to be correlated with low back pain during pregnancy [26]. However, it is not well established to evaluate comprehensive physical fitness during pregnancy with objective measurements [27]. Given that the changes of physical fitness level and the morbidity of low back pain along the entire pregnant course are still obscure, it is very difficuilt for most current exercise programs to accurately and specifically address low back pain at different stages of gestational stages.

Our team initially constructed a physical fitness test system for pregnant women which includes physique, physiological function and physical quality and have 23 component tests. These 23 component tests were constructed through three rounds of expert discussion with references to the physiological characteristics of pregnant women, exercise guidelines, exercise contraindications, exercise needs and existing physical fitness tests of people with limited exercise and elderly people. 60 participants completed all the tests without complaints or discomfort reported [28]. This evaluation system is scientific, practical and safe, and can be used as an evaluation tool for physical fitness during pregnancy.

Using this evaluation system, this study aimed to evaluate the physical fitness of pregnant women objectively, investigate pregnancy-related low back pain based on Oswestry Disability Index (ODI) [29] and explore the correlation between the low back pain and those 23 component tests in the physical fitness evaluation system. On one hand, this study provided methodological support for objective measurement of physical fitness for pregnant women. On the other hand, this preliminary exploration of relationship between physical fitness and low back pain during pregnancy laid a foundation for the research on the correlation and causality between physical fitness and other common symptoms during pregnancy, and also provided an objective reference for scientific exercise strategy for pregnant women.

## 2 Materials and methods

### 2.1 Study design

**Fig 1:**
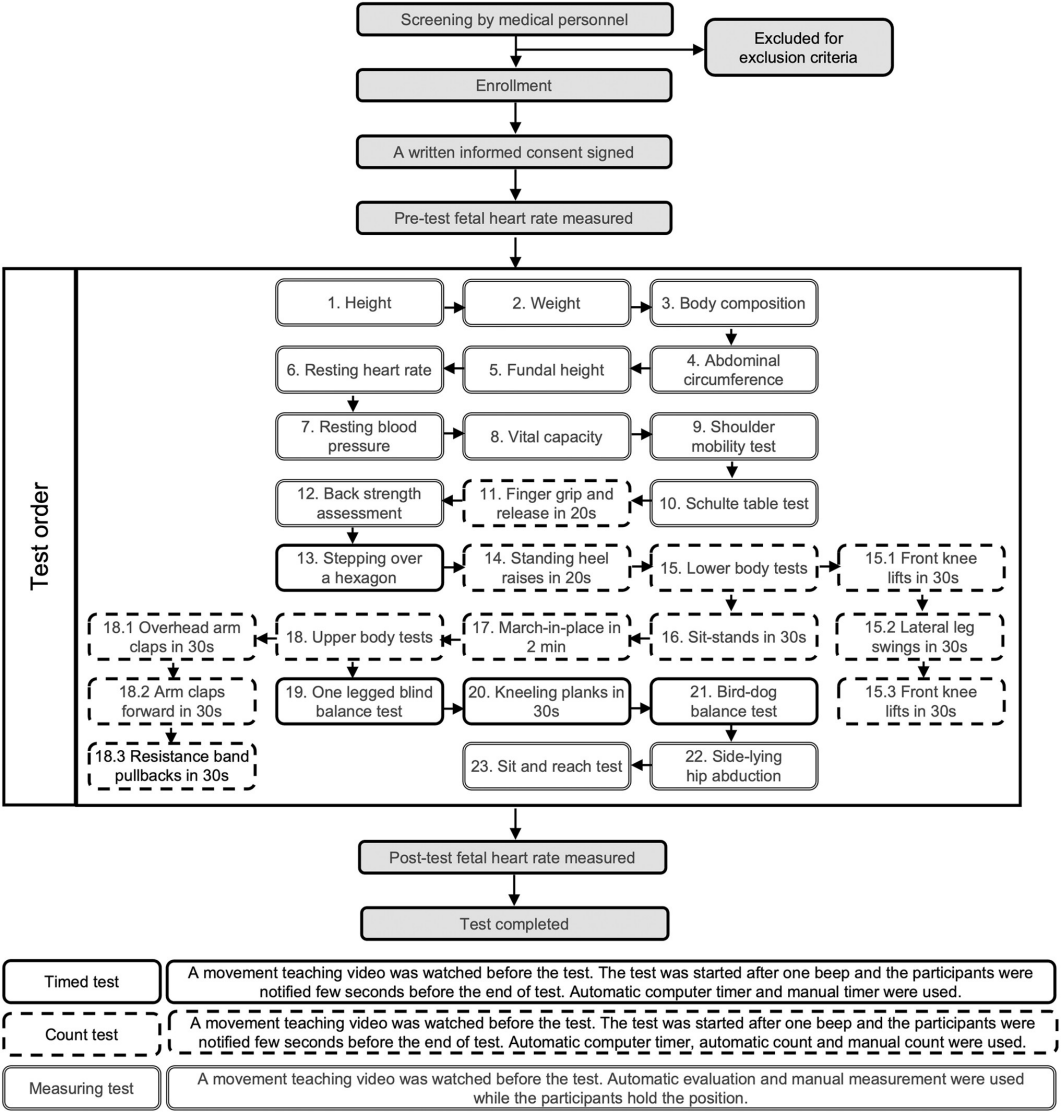
Flow chart of study participants.

The present study is a part of a project about correlation between objective physical fitness during pregnancy and several common pregnancy-related symptoms including low back pain. The physical fitness evaluation we used here is a test system for pregnant women mentioned above [28]. More details is displayed in Fig 1. To be scientific and reliable, all the participants performed the tests under the guidance of a computerized program with a video showing the standardized movement for every test, an accurate test timer and the same time interval between tests. A professional assistant and a doctor presenting were responsible for safety issues and collecting the test results.

Meanwhile all participants rated their low back pain from 0 to 5 compared with non-pregnant state and the correlation between low back pain and physical fitness was analyzed.

This study was approved by research ethics committee of Capital University of Physical Education and Sports (201910012).

### 2.2 Participants

A total of 180 participants were recruited and classified into mid-gestation group (14–28 weeks) and late-gestation group (28–37 weeks) according to their gestational week on the test day. 84 pregnant women (44 in mid-gestation and 40 in late gestation) were enrolled in July 2021 through Shanxi maternity and child health hospital (Shanxi, China) and 96 pregnant women (57 in mid-gestation and 39 in late gestation) were enrolled in September 2021 through Shandong provincial hospital east center (Shandong, China).

They were evaluated to meet the following criteria: (1) age between 22–40 years old; (2) gestation between 14–37 weeks with single fetus; (3) both pregnant women and fetus in health condition according to prenatal examination; (4) no assisted reproductive technology applied; (5) no smoking or alcohol habits; (6) no history of premature delivery, recurrent abortion, or fetus growth restriction; (7) no cardiopathy, hypertension and cerebrovascular disease history; (8) no pregnancy complications including premature rupture of membranes, polyhydramnios or oligohydramnios, fetal malformations and so on; (9) no dyskinesia or other exercise contraindications. In addition, to confirm that the participants have the ability to complete all tests safely in the assessment, we excluded the participants who had self-scaled low back pain intensity more than 3 based on the Oswestry Disability Index (ODI) [29]. The enrollment of participants whose pain intensity was 3 was determined after medical evaluation and discussion with the doctor and participants.

All the participants signed a written informed consent after being informed our study aims and procedures. All the data and personal information of participants were identified by numbers, and the personal information of the subjects will not be disclosed.

### 2.3 Self-reported low back pain

The participants were asked to scale the low back pain they suffered during pregnancy in comparison with their non-pregnancy state. They rated the intensity of their low back pain as “0 = no pain”, “1 = very mild pain”, “2 = moderate pain”, “3 = fairly severe pain”, “4 = very severe pain”, “5 = the worst imaginable pain” as the Oswestry Disability Index (ODI) [29].

### 2.4 Physical fitness assessment

All the participants were informed that the physical fitness assessment lasted around 30 minutes. For every component test, there was a teaching video showing the standard movement and the participants were asked to follow the instructions repeating the movement. The repetition in certain duration and/or time spent to complete movement were recorded. Accompanying with medical staff, the participants could terminate the assessment immediately if they felt uncomfortable. They were instructed to wear a wristband with a heart rate sensor. The assessment was started once the heart rate sensor was connected to the heart rate monitor and their basic information including name, age, height, gestation week was imported into the database in the computer.

A series of tests were performed in the order shown in Table 1. Instruction and equipment used for every test were attached below. The participants were allowed to take a break after all the test completion and asked for the assessment feedback. They also had post-test fetal heart rate collected when their heart rate and breath back to normal.

**Table 1:** Workflow of the physical fitness assessment.

Test order	Test name	Movement description	Test goal	Duration (second)	Equipment
**1**	Height	stand straight with bare feet	Longitudinal growth	30	A height meter
**2**	Weight	stand on the flatform with bare feet and push the handle till the result displayed	Horizontal growth	30	Inbody composition analyzer (InBody370)
**3**	Body composition		% of body fat	180	
**4**	Abdominal circumference	lay on the exam table after urination	Fetal growth	30	A measuring tape and an exam table
**5**	Fundal height			30	
	Fetal heart rate monitor before test				
**6**	Resting heart rate	have upper arm wrapped by a digital sphygmomanometer at heart level quietly till result displayed	Cardiovascular function	60	A digital sphygmomanometer
**7**	Resting blood pressure				
**8**	Vital capacity	inhale as deeply as possible and then exhale fully in one-time	Pulmonary ventilatory function	15	Hand-held spirometer (LK-T2016)
**9**	Shoulder mobility test	bend one elbow above and the other below the shoulder to get both fists close to each other behind the body to measure the distance between fists	Mobility of shoulders	15	A 50cm ruler
**10**	Schulte table test	find, read out and click all numbers in an ascending order of 16 numbers randomly arranged in a 4×4 table to record the time spent	Attention and neuromuscular Coordination	10	An app of Schulte table test
**11**	Finger grip and release in 20s	make tight fists and releases in 20s to record repetitions	Speed of finger movement	20	A stopwatch
**12**	Back strength assessment	pull up the handlebar without elbows and knees bent or backward fall standing on the chassis of a back dynamometer	Strength of back muscle group	30	back dynamometer (JY-BLJ)
**13**	Stepping over a hexagon	step over every line of a hexagon without body turning around and step back to the center in a clockwise order to record time spent	Multidirectional exercise performance	30	A roll of floor tape, a measuring tape, a stopwatch
**14**	Standing heel raises in 20s	raise heels off and lower down to the floor for 20s to record repetitions	Movement speed of lower limbs	20	A stopwatch
**15**	Lower body tests				
**15.1**	Front knee lifts in 30s	lift a knee to the hip level and release on 30s to record repetitions	Endurance of lower limbs’ muscle group	60	A stopwatch
**15.2**	Lateral leg swings in 30s	swing a leg out forming 45° with body and swing back in 30s for each leg to record repetitions			A stopwatch and a chair
**15.3**	Standing glute kickbacks in 30s	lift a leg behind to touch glute and lower back in 30s for each leg to record repetitions			
**16**	Sit-stands in 30s	stand up quickly and stably from a chair and sit back for 30s to record repetitions	strength of muscle group in lower limbs	30	
**17**	March-in-place in 2 min	march in place for 2min with lifted ankle higher than mid-calf of the supporting leg to record step repetitions	Cardiovascular function	120	A stopwatch
	1min rest				
**18**	Upper body tests				
**18.1**	Overhead arm claps in 30s	clap hands over head and lower back to the shoulder height for 30s to record repetitions	Endurance of upper limbs’ muscle group	30	A stopwatch
**18.2**	Arm claps forward in 30s	clap hands forward with elbow straight and abduct arms at shoulder height for 30s to record repetitions		30	
**18.3**	Resistance band pullbacks in 30s	hold two ends of a 10-pound resistance band that is fixed in the middle at shoulder height, pull them back to elbow bent at 90° and release at shoulder height for 30s to record repetitions	Strength and endurance of upper limbs’ muscle group	30	A stopwatch, a resistance band (10lb) and a resistance band holder
**19**	One legged blind balance test	hold the standing position with one foot off the ground and closed eyes as long as possible to record the time duration till maximum 30s	Proprioception and static balance without vision	60	A stopwatch
**20**	Kneeling plank	hold the position lying down with elbows and knees on the mat with raised calves and straight back for as long as possible to record the time duration till maximum 30s	Strength of the core muscle group	30	A yoga mat and a stopwatch
**21**	Bird-dog balance test	hold the bird-dog position with raised an arm overhead and contralateral leg lifted for as long as possible to record the time duration till maximum 30s	Ability to maintain body balance	60	
**22**	Side-lying hip abduction	lie down on their side on a yoga mat and have the upper leg abducted as far as they can to measure the angle between both legs	flexibility of hips	60	universal protractor (187–153, 0-500mm)
**23**	Sit and reach test	Lean forward slowly at the hips to push the cursor by their fingertips with straight legs sitting on a testing mat		30	Sit and Reach Flexibility Assessment Tester (wi99894 China)
	Test feedback				
	Fetal heart rate monitor after test				

### 2.5 Statistical analyses

Statistical analyses were performed using IBM SPSS Statistics for Windows (version 26.0, IBM Corp., Armonk, NY, USA) and the statistical significant was set as P<0.05. Data from individuals with incomplete results were excluded for analysis. All the data were presented as mean ± standard deviation (SD). Kolmogorov–Smirnov test was used to evaluate the normality of data of every test. Differences of variables between mid-gestation and late-gestation group were conducted by independent sample T test for normal distributed data and Mann-Whitney tests for non-normal distributed data. The relationships between low back pain with all physical fitness component tests were determined by spearman correlation analysis.

## 3 Results

### 3.1 Clinical anthropometric characteristics of participants

180 participants were completed the assessment as our final samples including 101 participants in mid-gestational stage and 79 participants in late-gestational stage. The anthropometric and clinical characteristics of participants were shown in Table 2. With the fetal development, the weight (P<0.01), body mass index (BMI) (P<0.01), body fat (P<0.01), abdominal circumference (P<0.01), fundal height (P<0.01) and resting heart rate (P<0.01) of participants in late-gestation were significantly higher than those in mid-gestation. However, there was no significant difference in age, height, blood pressure and fetal heart rate between mid-gestation and late-gestation groups. Additionally, no difference in fetal heart rate before and after assessment indicates that our physical fitness assessment was safe for the participants and their babies.

**Table 2:** Clinical anthropometric characteristics of participants (N = 180).

	Mid-gestation (N = 101)	Late-gestation (N = 79)	Total (N = 180)
Age	30.41±3.35	29.47±3.46	29.99±3.42
Gestational week	20.94±3.43	31.88±2.20**	25.74±6.19
Height (cm)	162.65±4.86	162.99±5.91	162.80±5.33
Weight (kg)	63.13±8.86	68.99±8.56**	65.70±9.19
BMI (kg/cm^2^)	24.00±3.28	25.99±2.71**	24.87±3.19
Body fat (%)	31.22±5.15	33.24±4.38**	32.11±4.92
Abdominal circumference (cm)	88.38±8.15	97.96±6.40**	92.58±8.82
Fundal height (cm)	20.28±3.40	30.06±3.02**	24.58±5.84
Heart rate (bpm)	89.57±11.93	95.20±12.02**	92.04±12.26
Blood systolic pressure (mmHg)	107.31±12.09	109.41±16.35	108.23±14.11
Blood diastolic pressure (mmHg)	67.57±9.80	66.58±8.42	67.14±9.20
Fetal heart rate (pre-test) (bpm)	145.27±6.68	144.97±6.21	145.14±6.46
Fetal heart rate (post-test) (bpm)	145.46±6.42	145.38±7.44	145.42±6.87

Data are presented as mean ± SD,

*p<0.05,

**p<0.01 mid-gestation vs late gestation

Abbreviation: BMI = body mass index

### 3.2 Self-reported low back pain during pregnancy

The level of low back pain during pregnancy was self-rated by the participants and shown in Table 3. Generally, 25% of all participants did not have low back pain at all (pain intensity = 0). 57.78% and 16.67% of participants experienced very mild pain that did not affect daily life (pain intensity = 1) and moderate pain that can be tolerated during daily life (pain intensity = 2), respectively (greater score means more severe pain). Only 1 participant suffered from fairly severe pain that may affect daily life (pain intensity = 3). In comparison of pain intensity of participants during middle and late gestations, the proportion of participants who have very mild pain was decrease, while the proportion of those who have no pain and moderate pain were increased indicating that some participants were adopted to the increasing abdominal load but it was even worse in some cases. In addition, the mean pain intensity during mid-gestion and late gestation was 0.91±0.62 and 0.95±0.71, respectively, indicating that low back pain happens throughout the pregnancy course.

**Table 3:** Self-reported low back pain during pregnancy.

Low back pain			Mid-gestation (N = 101)	Late-gestation (N = 79)	Total (N = 180)
Pain intensity	0 (no pain)	N(%)	23(22.77%)	22(27.85%)	45(25%)
	1 (very mild pain)	N(%)	65(64.36%)	39(49.37%)	104(57.78%)
	2 (moderate pain)	N(%)	12(11.88%)	18(22.78%)	30(16.67%)
	3 (fairly severe pain)	N(%)	1(0.99%)	0(0)	1(0.55%)
Average pain intensity			0.91±0.62	0.95±0.71	0.93±0.66

### 3.3 Objective assessed physical fitness during pregnancy

All parameters in physical fitness assessment were displayed in Table 4. Compared with women in mid-gestational stage, those in late gestation had significant weaker back strength (p<0.05) and less resistance band pullbacks in 30s (p<0.05), indicating that the core muscle strength of pregnant women in late-gestation was lower than that in mid-gestation. In addition, participants in late-gestation had less stretching in sit-and-reach test (p<0.01), shorter duration in left-legged blind balance test (p<0.05) and weaker bird-dog balance with right arm and left leg (p<0.05) suggesting that the flexibility and balance of pregnant women in late-gestation was poorer than that in mid-gestation. For the remaining tests, there was no significant difference between middle and late gestations.

**Table 4:** Comparison between objectively measured physical fitness during mid-gestation and late-gestation.

	Mid-gestation (N = 101)	Late-gestation (N = 79)	Total (N = 180)
Vital capacity (ml)	2631.97±609.88	2594.75±595.84	2615.63±602.36
2 min march-in-place (steps)	191.12±20.64	190.05±21.64	190.65±21.03
Kneeling plank (s)	29.35±2.52	26.32±8.70	28.02±6.23
Back strength assessment (kg)	32.09±10.22	28.59±9.07*	30.56±9.86
30s sit-stands	12.49±2.51	11.78±2.84	12.18±2.67
Schulte table test (s)	4.33±0.87	4.50±0.92	4.40±0.89
20s finger grip and release	29.74±7.83	30.82±8.52	30.22±8.13
20s standing heel raises	15.53±4.09	15.38±4.35	15.47±4.20
30s overhead arm claps	23.50±4.16	23.01±3.96	23.28±4.07
30s arm claps forward	24.11±3.65	23.25±3.70	23.73±3.69
30s resistance band pullbacks	22.87±4.87	21.22±4.59*	22.14±4.81
30s left front knee lifts	24.51±5.62	23.76±4.69	24.18±5.23
30s right front knee lifts	23.59±5.48	22.99±4.33	23.33±5.01
30s left lateral leg swings	25.77±6.80	23.95±5.93	24.97±6.48
30s right lateral leg swings	24.83±6.20	23.76±5.19	24.36±5.79
30s left standing glute kickbacks	24.63±6.51	24.49±6.06	24.57±6.30
30s right standing glute kickbacks	23.89±5.49	23.27±5.51	23.62±5.49
Left shoulder mobility test (cm)	12.51±6.33	12.73±6.63	12.60±6.45
Right shoulder mobility test (cm)	9.47±5.29	9.99±5.91	9.70±5.56
Sit and reach test (cm)	-1.80±7.63	-7.46±9.63**	-4.28±8.99
Left side-lying hip abduction (degree)	79.93±15.96	78.35±16.74	79.23±16.28
Right side-lying hip abduction (degree)	82.78±17.09	81.77±15.54	82.34±16.39
Stepping over a hexagon (s)	25.86±4.50	26.39±3.72	26.09±4.17
Left-legged blind balance test (s)	7.30±8.04	4.78±4.20*	6.20±6.73
Right-legged blind balance test (s)	5.56±4.87	4.85±5.03	5.25±4.94
Left bird-dog balance (s)	25.17±8.46	24.45±8.93	24.85±8.65
Right bird-dog balance (s)	26.25±7.22	23.43±9.15*	25.02±8.22

*p<0.05,

**p<0.01 mid-gestation vs late gestation

### 3.4 Correlations between low back pain and physical fitness during pregnancy

The coefficient of spearman correlation between self-reported low back pain with every component of physical fitness assessment were shown in Table 5. Among all these component tests, the low back pain was negatively associated with standing heel raises in 20s (p<0.01) and standing glute kickbacks in 30s (left, p<0.01; right, p<0.05). During mid-gestation, the low back pain was negatively correlated with standing glute kickbacks in 30s (both left and right, p<0.05).

**Table 5:** Correlations between low back pain and physical fitness during pregnancy.

	Mid-gestation (N = 101)	Late-gestation (N = 79)	Total (N = 180)
Height (cm)	-0.032	-0.026	-0.031
Weight (kg)	-0.083	-0.083	-0.042
BMI (kg/m^2^)	-0.069	0.012	-0.016
Body fat (%)	0.017	0.084	0.037
Abdominal circumference (cm)	0.083	-0.004	0.055
Fundal height (cm)	-0.056	-0.117	-0.040
Heart rate (bpm)	0.090	0.087	0.081
Blood systolic pressure (mmHg)	-0.044	-0.127	0.036
Blood diastolic pressure (mmHg)	-0.007	0.157	0.060
Vital capacity (ml)	0.125	-0.062	0.038
2 min march-in-place (steps)	0.065	-0.103	-0.014
Kneeling plank (s)	0.022	-0.016	-0.010
Back strength assessment (kg)	-0.100	0.101	-0.013
30s sit-stands	-0.024	-0.085	-0.065
Schulte table test (s)	0.096	-0.218	-0.049
20s finger grip and release	-0.135	0.026	-0.052
20s standing heel raises	-0.172	-0.214	-0.196**
30s overhead arm claps	-0.098	0.034	-0.036
30s arm claps forward	-0.144	0.050	-0.049
30s resistance band pullbacks	-0.138	0.137	-0.010
30s left front knee lifts	-0.149	-0.107	-0.126
30s right front knee lifts	-0.081	-0.086	-0.083
30s left lateral leg swings	-0.049	-0.158	-0.101
30s right lateral leg swings	-0.062	-0.104	-0.077
30s left standing glute kickbacks	-0.216*	-0.162	-0.196**
30s right standing glute kickbacks	-0.203*	-0.167	-0.184*
Left shoulder mobility test (cm)	-0.040	-0.094	-0.061
Right shoulder mobility test (cm)	-0.026	-0.050	-0.329
Sit and reach test (cm)	-0.032	0.041	-0.040
Left side-lying hip abduction (degree)	0.128	-0.016	0.053
Right side-lying hip abduction (degree)	0.167	-0.076	0.046
Stepping over a hexagon (s)	-0.117	0.111	-0.013
Left-legged blind balance test (s)	0.087	0.090	0.089
Right-legged blind balance test (s)	0.124	0.023	0.070
Left bird-dog balance (s)	-0.019	-0.002	-0.012
Right bird-dog balance (s)	0.037	0.107	0.062

*p<0.05,

**p<0.01 significantly correlated with low back pain

## 4 Discussion

In this study, physical fitness and low back pain were evaluated in 180 pregnant women and the correlations between low back pain and tests of physical fitness evaluation system including cardiorespiratory fitness, muscular strength, endurance, speed and body flexibility, balance were explored. As the main results, the physical fitness of pregnancy women was declined as seen weaker core strength, poorer flexibility, and worse balance in late gestation. Low back pain is commonly experienced during pregnancy. Less low back pain was correlated with greater lower limb motor function. Specifically, low back pain was negatively correlated with standing heel raises in 20s and left and right standing glute kickbacks in 30s.

Low back pain is one of common symptoms during pregnancy [26] as indicated that up to 75% of participants have low back pain in our study. It usually begins in the second trimester and continues in the remaining pregnancy course [6]. Consistently, our study shows that 77.23% of mid-gestational women and 72.15% of late-gestational women experience low back pain. As pregnancy progresses, the pelvis is anterior tilted gradually to compensate the center of gravity shift due to the enlarging abdomen and hormonal changes which accumulates extra load on lumbar spines inducing low back pain [6]. Additionally, lumbar muscles remain contracted to compensate the weak strength of abdominal muscles because of enlarging gravid uterus which contributes low back pain [30]. The average intensity of the low back pain in the participants during mid-gestation and late gestation was 0.91±0.62 and 0.95±0.71, respectively. The pain intensity in late gestation was slightly higher than that in mid-gestation without significant difference. It is consistent with published results [25, 26]. A probability is that pregnant women having more severe low back pain were excluded in our study because of safety consideration.

Pregnant women with low back pain usually have troubles with basic activities of daily living such as walking, standing up from chair and crunching [26, 31]. Our study designed a series of specific motions that are fundamental for daily activities to evaluate physical fitness during pregnancy and compared them between mid-gestation and late-gestation regarding cardiorespiratory fitness, muscular strength, endurance, speed and body flexibility, balance. In all motion tests, similar vital capacity and similar steps in 2 minutes March-in-place test show no difference in cardiorespiratory function between participants in mid-gestation and late gestation. In addition, they have comparable performance in Schulte table test, 20s finger grip and release test, 20s standing heel raise test and stepping over a hexagon indicating that speed ability and agility are not affected as the pregnancy processes. However, participants in late gestation have significant weaker back strength and significant less resistance band pullbacks in 30 seconds. These results suggest that the core muscle strength and endurance of pregnant women in late-gestation was weaker than those in mid-gestation. It seems that, increased relaxin release, a common reason inducing low back pain [32], affects the core skeletal muscular system. In addition, participants in late gestation have poorer flexibility and balance as indicated by less stretching distance in sit-and-reach test, shorter duration in left legged blind balance test and weaker bird dog balance with raised right arm and left leg. Cortell‐Tormo JM et al. suggested that improvements in muscular system and balance probably help females experience less low back pain [33]. Combined this finding and the differences between mid-gestation and late gestation in our results, it is clear that pregnancy-related physical fitness decrease may contribute to low back pain during the pregnant course.

In fact, it is already reported that greater overall physical fitness is associated with less bodily pain and lumbar pain during pregnancy [25]. Our study explored the correlation between low back pain and different types of physical fitness. Our result shows that, regardless the gestational week, low back pain in all participants is negatively correlated with standing heel raises in 20s and standing glute kickbacks (both left and right) in 30s. As we know, gluteal muscles and hamstring muscles are the primary muscles engaged in standing glute kickbacks [34], and gastrocnemius muscles and soleus muscle are responsible for standing heel raises [35]. The patients who have low back pain generally have weak gluteal medius [36] and chronic low back pain limits trunk-pelvis, pelvis-thigh coordination in sagittal plane as well as lower extremities during walking [37]. While heel raise and glute kickback in standing posture are commonly repeated in walking, our speculation is that pregnant women with low back pain have weaker lower back and limb kinematics, especially lumber-pelvis-thigh-calf coordination. The contracted and weakened lumber muscles not only contribute to low back pain as mentioned earlier, but also result in weakened musculoskeletal activities of hip, knees, and ankles which lead to poorer balance and flexibility subsequently.

Interestingly, when we considered the correlations in middle and late gestations separately, low back pain was correlated with standing glute kickbacks (both left and right) in 30s only during middle gestation. Standing glute kickback tests the rapid contraction capability and coordination of muscle groups in hips and back thighs that is relatively complicated and difficult for all the participants. Participants without low back pain in mid-gestation could do better than those with low back pain making it correlated in glute kickback test, however, participants in late gestation have more bulky body and complete kickback with more difficulty no matter whether low back pain is present or not. All the evidence suggest that pregnancy-related low back pain is correlated to weak physical fitness. Further studies are needed to explore preventive and therapeutic exercise treatments for low back pain during pregnancy, especially treatments improving ability of core and lower limbs.

Our study has limitations. Firstly, we have 180 participants for current study. The sample size could be larger. Secondly, all participants enrolled are pregnant. Only difference between mid-gestation and late gestation is analyzed. Participants in preconception and puerperium could also be recruited to evaluate change of physical fitness and low back pain because of pregnancy and delivery since low back pain continues in some women in the postpartum period.

## 5 Conclusion

Low back pain is in high prevalence throughout the entire pregnant course. The pregnant women are prone to have weaker strength of core muscle groups and poorer flexibility and balance along the pregnancy. In addition, their low back pain was negatively correlated to strength of back muscle groups of lower limbs.

## Supporting information

S1 Checklist STROBE statement—Checklist of items that should be included in reports of observational studies. (DOCX)

## References

[1] Berber MA, Satilmis IG. Characteristics of Low Back Pain in Pregnancy, Risk Factors, and Its Effects on Quality of Life. Pain Manag Nurs. 2020;21(6):579–86. Epub 2020/06/24. doi: 10.1016/j.pmn.2020.05.001 . 32571670

[2] Mota MJ, Cardoso M, Carvalho A, Marques A, Sa-Couto P, Demain S. Women’s experiences of low back pain during pregnancy. J Back Musculoskelet Rehabil. 2015;28(2):351–7. Epub 2014/10/02. doi: 10.3233/BMR-140527 . 25271197

[3] Fruscalzo A, Cocco P, Londero AP, Gantert M. Low Back Pain during Pregnancy and Delivery Outcomes. Z Geburtshilfe Neonatol. 2022;226(2):104–11. Epub 2021/08/26. doi: 10.1055/a-1553-4856 . 34433210

[4] Rodriguez-Ayllon M, Acosta-Manzano P, Coll-Risco I, Romero-Gallardo L, Borges-Cosic M, Estevez-Lopez F, et al. Associations of physical activity, sedentary time, and physical fitness with mental health during pregnancy: The GESTAFIT project. J Sport Health Sci. 2021;10(3):379–86. Epub 2021/05/25. doi: 10.1016/j.jshs.2019.04.003 34024352 PMC8167327

[5] Borg-Stein J, Dugan SA. Musculoskeletal disorders of pregnancy, delivery and postpartum. Phys Med Rehabil Clin N Am. 2007;18(3):459–76, ix. Epub 2007/08/07. doi: 10.1016/j.pmr.2007.05.005 . 17678762

[6] Casagrande D, Gugala Z, Clark SM, Lindsey RW. Low Back Pain and Pelvic Girdle Pain in Pregnancy. J Am Acad Orthop Surg. 2015;23(9):539–49. Epub 2015/08/15. doi: 10.5435/JAAOS-D-14-00248 . 26271756

[7] Han IH. Pregnancy and spinal problems. Curr Opin Obstet Gynecol. 2010;22(6):477–81. Epub 2010/10/12. doi: 10.1097/GCO.0b013e3283404ea1 . 20930629

[8] To WW, Wong MW. Factors associated with back pain symptoms in pregnancy and the persistence of pain 2 years after pregnancy. Acta Obstet Gynecol Scand. 2003;82(12):1086–91. Epub 2003/11/18. doi: 10.1046/j.1600-0412.2003.00235.x . 14616251

[9] Ho SS, Yu WW, Lao TT, Chow DH, Chung JW, Li Y. Effectiveness of maternity support belts in reducing low back pain during pregnancy: a review. J Clin Nurs. 2009;18(11):1523–32. Epub 2009/06/06. doi: 10.1111/j.1365-2702.2008.02749.x . 19490291

[10] Reyhan AC, Dereli EE, Colak TK. Low back pain during pregnancy and Kinesio tape application. J Back Musculoskelet Rehabil. 2017;30(3):609–13. Epub 2016/12/31. doi: 10.3233/BMR-160584 . 28035911

[11] Xue X, Yang X, Deng Z, Chen Y, Mao X, Tu H, et al. Effect of Kinesio taping on Pregnancy-related low back pain: A protocol for systematic review and meta-analysis. PLoS One. 2022;17(1):e0261766. Epub 2022/01/21. doi: 10.1371/journal.pone.0261766 35051196 PMC8775207

[12] Liddle SD, Pennick V. Interventions for preventing and treating low-back and pelvic pain during pregnancy. Cochrane Database Syst Rev. 2015;(9):CD001139. Epub 2015/10/01. doi: 10.1002/14651858.CD001139.pub4 26422811 PMC7053516

[13] Oliveira CB, Maher CG, Pinto RZ, Traeger AC, Lin CC, Chenot JF, et al. Clinical practice guidelines for the management of non-specific low back pain in primary care: an updated overview. Eur Spine J. 2018;27(11):2791–803. Epub 2018/07/05. doi: 10.1007/s00586-018-5673-2 . 29971708

[14] Knezevic NN, Candido KD, Vlaeyen JWS, Van Zundert J, Cohen SP. Low back pain. Lancet. 2021;398(10294):78–92. Epub 2021/06/12. doi: 10.1016/S0140-6736(21)00733-9 . 34115979

[15] Luomajoki HA, Bonet Beltran MB, Careddu S, Bauer CM. Effectiveness of movement control exercise on patients with non-specific low back pain and movement control impairment: A systematic review and meta-analysis. Musculoskelet Sci Pract. 2018;36:1–11. Epub 2018/04/10. doi: 10.1016/j.msksp.2018.03.008 . 29631119

[16] Gregg VH, Ferguson JE 2nd. Exercise in Pregnancy. Clin Sports Med. 2017;36(4):741–52. Epub 2017/09/10. doi: 10.1016/j.csm.2017.05.005 . 28886825

[17] Melzer K, Schutz Y, Boulvain M, Kayser B. Physical activity and pregnancy: cardiovascular adaptations, recommendations and pregnancy outcomes. Sports Med. 2010;40(6):493–507. Epub 2010/06/08. doi: 10.2165/11532290-000000000-00000 . 20524714

[18] Bacchi E, Bonin C, Zanolin ME, Zambotti F, Livornese D, Dona S, et al. Physical Activity Patterns in Normal-Weight and Overweight/Obese Pregnant Women. PLoS One. 2016;11(11):e0166254. Epub 2016/11/10. doi: 10.1371/journal.pone.0166254 27829017 PMC5102361

[19] Bull FC, Al-Ansari SS, Biddle S, Borodulin K, Buman MP, Cardon G, et al. World Health Organization 2020 guidelines on physical activity and sedentary behaviour. Br J Sports Med. 2020;54(24):1451–62. Epub 2020/11/27. doi: 10.1136/bjsports-2020-102955 33239350 PMC7719906

[20] Nascimento SL, Surita FG, Godoy AC, Kasawara KT, Morais SS. Physical Activity Patterns and Factors Related to Exercise during Pregnancy: A Cross Sectional Study. PLoS One. 2015;10(6):e0128953. Epub 2015/06/18. doi: 10.1371/journal.pone.0128953 26083416 PMC4470997

[21] Zhang Y, Dong S, Zuo J, Hu X, Zhang H, Zhao Y. Physical activity level of urban pregnant women in Tianjin, China: a cross-sectional study. PLoS One. 2014;9(10):e109624. Epub 2014/10/07. doi: 10.1371/journal.pone.0109624 25286237 PMC4186867

[22] Yan CF, Hung YC, Gau ML, Lin KC. Effects of a stability ball exercise programme on low back pain and daily life interference during pregnancy. Midwifery. 2014;30(4):412–9. Epub 2013/06/14. doi: 10.1016/j.midw.2013.04.011 . 23759131

[23] Fontana Carvalho AP, Dufresne SS, Rogerio de Oliveira M, Couto Furlanetto K, Dubois M, Dallaire M, et al. Effects of lumbar stabilization and muscular stretching on pain, disabilities, postural control and muscle activation in pregnant woman with low back pain. Eur J Phys Rehabil Med. 2020;56(3):297–306. Epub 2020/02/20. doi: 10.23736/S1973-9087.20.06086-4 . 32072792

[24] Sklempe Kokic I, Ivanisevic M, Uremovic M, Kokic T, Pisot R, Simunic B. Effect of therapeutic exercises on pregnancy-related low back pain and pelvic girdle pain: Secondary analysis of a randomized controlled trial. J Rehabil Med. 2017;49(3):251–7. Epub 2017/02/25. doi: 10.2340/16501977-2196 . 28233012

[25] Marin-Jimenez N, Acosta-Manzano P, Borges-Cosic M, Baena-Garcia L, Coll-Risco I, Romero-Gallardo L, et al. Association of self-reported physical fitness with pain during pregnancy: The GESTAFIT Project. Scand J Med Sci Sports. 2019;29(7):1022–30. Epub 2019/04/02. doi: 10.1111/sms.13426 . 30933387

[26] Morino S, Ishihara M, Umezaki F, Hatanaka H, Iijima H, Yamashita M, et al. Low back pain and causative movements in pregnancy: a prospective cohort study. BMC Musculoskelet Disord. 2017;18(1):416. Epub 2017/10/19. doi: 10.1186/s12891-017-1776-x 29037184 PMC5644197

[27] Romero-Gallardo L, Roldan Reoyo O, Castro-Pinero J, May LE, Ocon-Hernandez O, Mottola MF, et al. Assessment of physical fitness during pregnancy: validity and reliability of fitness tests, and relationship with maternal and neonatal health—a systematic review. BMJ Open Sport Exerc Med. 2022;8(3):e001318. Epub 2022/09/30. doi: 10.1136/bmjsem-2022-001318 36172399 PMC9511659

[28] Zhou L, Sun Z, Zheng R, et al. System construction of physical fitness index for pregnant women (孕妇体质测试指标体系的构建). Chinese Journal of Perinatal Medicine 2021;12: 677–681. [in Chinese].

[29] Fairbank JC, Pynsent PB. The Oswestry Disability Index. Spine (Phila Pa 1976). 2000;25(22):2940–52; discussion 52. Epub 2000/11/14. doi: 10.1097/00007632-200011150-00017 . 11074683

[30] Ireland ML, Ott SM. The effects of pregnancy on the musculoskeletal system. Clin Orthop Relat Res. 2000;(372):169–79. Epub 2000/03/30. doi: 10.1097/00003086-200003000-00019 . 10738426

[31] Close C, Sinclair M, Liddle D, Mc Cullough J, Hughes C. Women’s experience of low back and/or pelvic pain (LBPP) during pregnancy. Midwifery. 2016;37:1–8. Epub 2016/05/25. doi: 10.1016/j.midw.2016.03.013 . 27217231

[32] Tong MH, Mousavi SJ, Kiers H, Ferreira P, Refshauge K, van Dieen J. Is There a Relationship Between Lumbar Proprioception and Low Back Pain? A Systematic Review With Meta-Analysis. Arch Phys Med Rehabil. 2017;98(1):120–36 e2. Epub 2016/06/19. doi: 10.1016/j.apmr.2016.05.016 . 27317866

[33] Cortell-Tormo JM, Sanchez PT, Chulvi-Medrano I, Tortosa-Martinez J, Manchado-Lopez C, Llana-Belloch S, et al. Effects of functional resistance training on fitness and quality of life in females with chronic nonspecific low-back pain. J Back Musculoskelet Rehabil. 2018;31(1):95–105. Epub 2017/08/23. doi: 10.3233/BMR-169684 . 28826168

[34] Stevens BM, Nichols BR, Doty HI, Korak JA. Muscle Activation Patterns of the Proximal Medial and Distal Biceps Femoris and Gluteus Maximus Among 6 Hip Extension and Knee Flexion Exercises in Trained Women. Int J Exerc Sci. 2022;15(1):1179–89. Epub 2022/08/23. 35989703 10.70252/DWMB8342 PMC9362892

[35] Ugbolue UC, Yates EL, Ferguson K, Wearing SC, Gu Y, Lam WK, et al. Electromyographic Assessment of the Lower Leg Muscles during Concentric and Eccentric Phases of Standing Heel Raise. Healthcare (Basel). 2021;9(4). Epub 2021/05/01. doi: 10.3390/healthcare9040465 33919959 PMC8070905

[36] Kameda M, Tanimae H, Kihara A, Matsumoto F. Does low back pain or leg pain in gluteus medius syndrome contribute to lumbar degenerative disease and hip osteoarthritis and vice versa? A literature review. J Phys Ther Sci. 2020;32(2):173–91. Epub 2020/03/12. doi: 10.1589/jpts.32.173 32158082 PMC7032979

[37] Ebrahimi S, Kamali F, Razeghi M, Haghpanah SA. Comparison of the trunk-pelvis and lower extremities sagittal plane inter-segmental coordination and variability during walking in persons with and without chronic low back pain. Hum Mov Sci. 2017;52:55–66. Epub 2017/01/26. doi: 10.1016/j.humov.2017.01.004 . 28119210

