# Supplementary material for: The correlation between pregnancy-related low back pain and physical fitness evaluated by an index system of maternal physical fitness test
Source: PLoS One. 2023 Dec 21;18(12):e0294781. doi: 10.1371/journal.pone.0294781 (PMC10734947; doi:10.1371/journal.pone.0294781)
Supplement: S1 Checklist — (DOCX) [file pone.0294781.s001.docx]

STROBE Statement—checklist of items that should be included in reports of observational studies

|  | Item No. | Recommendation | Page  No. | Relevant text from manuscript |
| --- | --- | --- | --- | --- |
| **Title and abstract** | 1 | (*a*) Indicate the study’s design with a commonly used term in the title or the abstract | 1 | The correlation between…and... |
|  |  | (*b*) Provide in the abstract an informative and balanced summary of what was done and what was found | 2 | LBP was strongly negatively correlated to strength of back muscle groups of lower limbs. |
| Introduction | | | |  |
| Background/rationale | 2 | Explain the scientific background and rationale for the investigation being reported | 3,4 | Given that the changes of physical fitness level and the morbidity of low back pain along the entire pregnant course are still obscure… |
| Objectives | 3 | State specific objectives, including any prespecified hypotheses | 4 | aims to evaluate the physical fitness … objectively …, investigate pregnancy-related low back pain … and explore the correlation between the low back pain and … |
| Methods | | | |  |
| Study design | 4 | Present key elements of study design early in the paper | 4,5 | The physical fitness assessment we used here is… Meanwhile all participants rated their low back pain …and the correlation between … |
| Setting | 5 | Describe the setting, locations, and relevant dates, including periods of recruitment, exposure, follow-up, and data collection | 5 | 84 pregnant women …enrolled in July 2021 through … hospital (Shanxi, China) and 96 pregnant women ..September 2021 through … (Shandong, China). |
| Participants | 6 | (*a*) *Cohort study*—Give the eligibility criteria, and the sources and methods of selection of participants. Describe methods of follow-up  *Case-control study*—Give the eligibility criteria, and the sources and methods of case ascertainment and control selection. Give the rationale for the choice of cases and controls  *Cross-sectional study*—Give the eligibility criteria, and the sources and methods of selection of participants | 5,6 | They were evaluated to meet the following criteria: … |
|  |  | (*b*) *Cohort study*—For matched studies, give matching criteria and number of exposed and unexposed  *Case-control study*—For matched studies, give matching criteria and the number of controls per case | N/A |  |
| Variables | 7 | Clearly define all outcomes, exposures, predictors, potential confounders, and effect modifiers. Give diagnostic criteria, if applicable | 6,7 | For every component test, there was a teaching video showing the standard movement and the participants were asked to follow the instructions repeating the movement. … |
| Data sources/ measurement | 8* | For each variable of interest, give sources of data and details of methods of assessment (measurement). Describe comparability of assessment methods if there is more than one group | 6,7 | They rated the intensity of their low back pain…  a teaching video showing the standard movement and the participants were asked to follow the instructions repeating the movement. The repetition in certain duration and/or time spent to complete movement were recorded. |
| Bias | 9 | Describe any efforts to address potential sources of bias |  |  |
| Study size | 10 | Explain how the study size was arrived at | 5 | A total of 180 participants were recruited … |

Continued on next page

| Quantitative variables | 11 | Explain how quantitative variables were handled in the analyses. If applicable, describe which groupings were chosen and why | 8 | All the data were presented as mean ± standard deviation (SD). |
| --- | --- | --- | --- | --- |
| Statistical methods | 12 | (*a*) Describe all statistical methods, including those used to control for confounding | 8 | unpaired T test, partial correlation |
|  |  | (*b*) Describe any methods used to examine subgroups and interactions | 8 | Differences of variables between … unpaired T test  The relationships between…determined by partial correlation after adjusted for gestational week |
|  |  | (*c*) Explain how missing data were addressed | 8 | Data from individuals with incomplete results were excluded for analysis. |
|  |  | (*d*) *Cohort study*—If applicable, explain how loss to follow-up was addressed  *Case-control study*—If applicable, explain how matching of cases and controls was addressed  *Cross-sectional study*—If applicable, describe analytical methods taking account of sampling strategy | N/A |  |
|  |  | (*e*) Describe any sensitivity analyses | N/A |  |
| Results | | | | |
| Participants | 13* | (a) Report numbers of individuals at each stage of study—eg numbers potentially eligible, examined for eligibility, confirmed eligible, included in the study, completing follow-up, and analysed | 5,9 | 180 participants were completed the assessment as our final samples including 101 participants in mid-gestational stage and 79 participants in late-gestational stage. |
|  |  | (b) Give reasons for non-participation at each stage | 8 | Data from individuals with incomplete results were excluded for analysis. |
|  |  | (c) Consider use of a flow diagram | 7 |  |
| Descriptive data | 14* | (a) Give characteristics of study participants (eg demographic, clinical, social) and information on exposures and potential confounders | 9 | The anthropometric and clinical characteristics of participants were shown in Table 2. |
|  |  | (b) Indicate number of participants with missing data for each variable of interest | N/A |  |
|  |  | (c) *Cohort study*—Summarise follow-up time (eg, average and total amount) | N/A |  |
| Outcome data | 15* | *Cohort study*—Report numbers of outcome events or summary measures over time | N/A |  |
|  |  | *Case-control study—*Report numbers in each exposure category, or summary measures of exposure | *9* | 180 participants were completed the assessment as our final samples including 101 participants in mid-gestational stage and 79 participants in late-gestational stage. |
|  |  | *Cross-sectional study—*Report numbers of outcome events or summary measures | N/A |  |
| Main results | 16 | (*a*) Give unadjusted estimates and, if applicable, confounder-adjusted estimates and their precision (eg, 95% confidence interval). Make clear which confounders were adjusted for and why they were included | 11,12 | The coefficient of partial correlation between self-reported low back pain with every component of physical fitness assessment were shown in Table 5 |
|  |  | (*b*) Report category boundaries when continuous variables were categorized | N/A |  |
|  |  | (*c*) If relevant, consider translating estimates of relative risk into absolute risk for a meaningful time period | N/A |  |

Continued on next page

| Other analyses | 17 | Report other analyses done—eg analyses of subgroups and interactions, and sensitivity analyses | N/A |  |
| --- | --- | --- | --- | --- |
| Discussion | | | | |
| Key results | 18 | Summarise key results with reference to study objectives | 13 | As the main results, the physical fitness of pregnancy women was declined as seen weaker core… |
| Limitations | 19 | Discuss limitations of the study, taking into account sources of potential bias or imprecision. Discuss both direction and magnitude of any potential bias | 16 | Our study has limitations. Firstly… |
| Interpretation | 20 | Give a cautious overall interpretation of results considering objectives, limitations, multiplicity of analyses, results from similar studies, and other relevant evidence | 13-15 | It usually begins … Consistently, our study shows that… |
| Generalisability | 21 | Discuss the generalisability (external validity) of the study results | 15 | Further studies are needed to explore preventive and therapeutic exercise treatments for low back pain during pregnancy, especially treatments improving ability of core and lower limbs. |
| Other information | |  | | |
| Funding | 22 | Give the source of funding and the role of the funders for the present study and, if applicable, for the original study on which the present article is based | N/A |  |

*Give information separately for cases and controls in case-control studies and, if applicable, for exposed and unexposed groups in cohort and cross-sectional studies.

**Note:** An Explanation and Elaboration article discusses each checklist item and gives methodological background and published examples of transparent reporting. The STROBE checklist is best used in conjunction with this article (freely available on the Web sites of PLoS Medicine at http://www.plosmedicine.org/, Annals of Internal Medicine at http://www.annals.org/, and Epidemiology at http://www.epidem.com/). Information on the STROBE Initiative is available at www.strobe-statement.org.
