# Supplementary material for: The correlation between pregnancy-related low back pain and physical fitness evaluated by an index system of maternal physical fitness test
Source: PLoS One. 2023 Dec 21;18(12):e0294781. doi: 10.1371/journal.pone.0294781 (PMC10734947; doi:10.1371/journal.pone.0294781)
Supplement: Attachment — Submitted filename: Response to Reviewers.docx [file pone.0294781.s002.docx]

PONE-D-23-11001

The correlation between pregnancy-related low back pain and physical fitness evaluated by an index system of maternal physical fitness test

PLOS ONE

Additional Editor Comments (if provided):

INTRODUCTION

1. A summary of the findings from prevıous studıes on the normal values of some of the physical fitness parameters (age and gender matched) assessed in this present study, wrıtten in a small paragraph withın the ıntroductıon, will be useful

Thank you for your suggestion. A summary of the physical fitness assessment construction is added at line 88-96 in the text. Actually, these parameters are included after initial reference to physical fitness tests for people with limited exercise and elderly people and further be modified based on physiological characteristics of pregnant women, exercise guidelines for pregnant women. The normal values from age-matched nonpregnant females are being collected in our ongoing project. They have more repetitions in a given period and longer duration of a given position hold compared with those pregnant women.

2. Few lines on the author’s perceived sıgnıfıcance of the study could also be ıncluded within the ıntroduction section

We have added few sentences in the end of introduction (line 97-105).

METHODS

PARTICPANTS

What was the sampling technique?

**Answer**: All the participants were recruited from two hospitals as follows: our equipment for physical fitness assessment was set up for 2 weeks in each hospital. During the two weeks, every pregnant woman coming for regular pregnancy check-ups has opportunity to participate as long as [1] screened by her obstetrician and gynecologist to meet criteria including (1) age between 22-40 years old; (2) gestation between 14-37 weeks with single fetus; (3) both pregnant women and fetus in health condition according to prenatal examination; (4) no assisted reproductive technology applied; (5) no smoking or alcohol habits; (6) no history of premature delivery, recurrent abortion, or fetus growth restriction; (7) no cardiopathy, hypertension and cerebrovascular disease history; (8) no pregnancy complications including premature rupture of membranes, polyhydramnios or oligohydramnios, fetal malformations and so on; (9) no dyskinesia or other exercise contraindications; [2] had self-scaled low back pain intensity no more than 3 based on the Oswestry Disability Index (ODI) to confirm that she had the ability to complete all tests safely; [3] agreed to participate after being informed our study aims and procedures. To simplified show participation of our study samples, we added a figure (Fig.1) displaying our flow chart of study participants.

Lıne 145-159; PHYSICAL FITNESS ASSSESSMENT

1. What the order of carryıng out the tests that were lısted ın Table 1? –was ıt serially as lısted in the table or randomly?

**Answer**: Yes, the tests were carried out in the order exactly as listed in table 1 column 1 as well as Figure 1.

2. Were the participants allowed any resting perıods ın between one test and the next one? What measure was put in place to prevent carry over effect of performing one test before the next one

**Answer**: Participants did not have resting period between tests except 1min rest after test #17 (March-in-place in 2 min) to avoid heart rate over 160bpm. Generally, the entire assessment containing 23 component tests was carried out with consecutive automatic computerized guidance (standard instruction, automatic timer, and computerized test pattern). For every test, a teaching video showing the standard movement and corresponding instructions was displayed and the participant started repeating the movement or hold the position after an automatic “beep” and stopped at another “beep”.

As mentioned earlier, the tests were carried out in the same order listed in table 1, for all participants. In this order, we arranged successive tests that have different primary muscles engaged to prevent the potential effect of one test on the next one. For example, #15, the three lower body tests, 15.1 front knee lifts in 30s primarily targets quadriceps femoris, 15.2 lateral leg swings in 30s targets tensor fasciae latae, gluteus medius and gluteus minimus, standing glute kickbacks in 30s targets the gluteus maximus.

3. How accurate are the scores obtained from this tests? How many repetitions of the test were carried out before recordıng the fınal output? Could ıt be once or three tımes with an average scores being calculated?

**Answer**: In our computer program for physical fitness assessment, the movement of participants was recognized so that movement repetitions or/and duration of holding the position were recorded automatically. They were also evaluated manually by a professional assistant to confirm their accuracy. Every test was carried out only once and its score was the final output.

4. Authors should provıde a reference for the procedure used for each of the test as carried out, perhaps in a separate column in Table 1

**Answer**: These 23 component tests were indeed constructed with references of physical fitness tests for people with limited exercise and elderly people. However, they were also modified based on physiological characteristics of pregnant women, exercise guidelines for pregnant women, exercise contraindications for pregnant women. These are already described in our previous publication (in Chinese), this study focuses on correlation between those with low back pain. Therefore, we do not include the test reference here.

[Note: HTML markup is below. Please do not edit.]

Reviewers' comments:

[NOTE: If reviewer comments were submitted as an attachment file, they will be attached to this email and accessible via the submission site. Please log into your account, locate the manuscript record, and check for the action link "View Attachments". If this link does not appear, there are no attachment files.]

While revising your submission, please upload your figure files to the Preflight Analysis and Conversion Engine (PACE) digital diagnostic tool, https://pacev2.apexcovantage.com/. PACE helps ensure that figures meet PLOS requirements. To use PACE, you must first register as a user. Registration is free. Then, login and navigate to the UPLOAD tab, where you will find detailed instructions on how to use the tool. If you encounter any issues or have any questions when using PACE, please email PLOS at figures@plos.org. Please note that Supporting Information files do not need this step.
