# Supplementary material for: The correlation between pregnancy-related low back pain and physical fitness evaluated by an index system of maternal physical fitness test
Source: PLoS One. 2023 Dec 21;18(12):e0294781. doi: 10.1371/journal.pone.0294781 (PMC10734947; doi:10.1371/journal.pone.0294781)
Supplement: Attachment — Submitted filename: response to reviewers.docx [file pone.0294781.s003.docx]

Ref: PONE-D-23-11001

Title: The correlation between pregnancy-related low back pain and physical fitness evaluated by an index system of maternal physical fitness test

**Reviewer #1:** This study analyses data from 180 pregnant women at various stages in pregnancy who complete a suite of fitness tests designed by the authors. The details surrounding design of the tests and application in pregnancy is detailed in a separate publication. 5. The prevalence of LBP reported by the women in the study (13% mid-gestation and 23% late-gestation).

There are some grammatical and typos throughout that would need to be addressed but overall the article is well written.

My main concerns are with the statistical analysis and reporting of results:

1. Descriptive statistics for numeric data are reported as mean +- SD without any consideration of outliers or skewness in the data across the variables measured. Were the distributions of the numeric variables examined for outliers or skewness? This should be detailed in the statistical analysis and reported. Were numeric data is strongly skewed it should be summarised using median and IQR and between groups tests would use the Mann-Whitney test.

**Answer:** thanks for this suggestion. We have done normality tests for all the data by Kolmogorov–Smirnov test as well as histogram and Q-Q plot. Most of the variables conform to normal distribution law. For consistency in the data description, we keep presenting the numeric data description in the most common way as Mean± SD. For data analysis, we have screened outliers based on scatter-plots, box-plots and Z-score results, then replaced the outliers with mean (data sets in normal distribution) or median (non-normal distribution). Differences of variables between mid-gestation and late-gestation group have been conducted by independent sample T test（normal distributed data） and Mann-Whitney tests (non-normal distributed data).

2. Multiple testing: Table 4 contains 27 variables which are examined using independent samples t-tests. With this many tests, the researchers run a considerable risk of Type 1 errors - that is rejecting a null hypothesis incorrectly. It would be expected to find at least one statistically significant p-value even if there were no differences in the population. Some consideration and adjustment for Type 1 errors due to multiple testing should be made, for example using a Bonferroni adjustment. This also holds for Table 5 as well with 36 different variables tested for association.

**Answer:** thank you very much for this suggestion. We sorted out the current physical fitness tests for pregnant women, mainly focusing on contraindications for exercise during pregnancy, combining or revising some existing testing systems, and paying less attention to factors such as the symptoms of lack of exercise, exercise demand, and test load during pregnancy. We believe that the goal of physical fitness testing during pregnancy is not only to understand physical fitness level, but also to set relevant standards through physical fitness level, to warn of adverse pregnancy symptoms caused by the decline of physical fitness level during pregnancy or possible adverse birth outcomes, so as to improve adverse pregnancy symptoms, optimize birth outcomes, alleviate birth fears, and enhance pregnancy experience.

Based on this, we try to construct a testing system for pregnant women's physical fitness. We finalized with 23 tests after 3 rounds of Delphi survey by 10 experts (7 maternity medical specialists and 3 exercise experts) from 47 preliminary tests. Thus, as the reviewer’s opinion, it is vulnerable to have a risk of Type 1 errors in our analysis.

Therefore, we considered using False Discovery Rate (FDR) adjustment to reduce the risk of type 1 error. However, On the premise that there are many tests and the relationship between tests and objectives is not clear, FDR adjustment for all the tests may greatly increases the risk of type 2 errors- that is accepting a null hypothesis incorrectly. For example in Table 5, FDR adjustment is indeed effective to reduce the risk of type 1 errors but also greatly increase the risk of type 2 errors since multiple physical fitness tests including Height, Weight, BMI, Vital capacity, Schulte table test and other tests may have tiny correlations with low back pain.

To address above problem, we have two solutions. The first solution is to streamline control variables: If we have enough data to prove that low back pain is related to the low back pain and muscular strength and dynamic machenical chain of posterior body, we can perform FDR adjustment on partial tests that are important factors to low back pain such as 20s standing heel raises, 30s left standing glute kickbacks, 30s right standing glute kickbacks, Back strength assessment, Kneeling plank (s), 30s resistance band pullbacks as shown in the following table. The final result is consistent with our paper.

|  | **Mid-gestation (N=101)** | **P**  **value** | **FDR（BH）** | **Late-gestation (N=79)** | **P**  **value** | **FDR（BH）** | **Total (N=180)** | **P**  **value** | **Rank** | **FDR（BH）** |
| --- | --- | --- | --- | --- | --- | --- | --- | --- | --- | --- |
| 20s standing heel raises | -0.1717 | 0.0861 | 0.0188 | -0.2136 | 0.0587 | 0.0063 | 0.1963** | 0.0082 | 1 | 0.0063 |
| 30s left standing glute kickbacks | -0.2158* | 0.0302 | 0.0063 | -0.1674 | 0.1404 | 0.0125 | 0.1956** | 0.0085 | 2 | 0.0125 |
| 30s right standing glute kickbacks | -0.2032* | 0.0415 | 0.0125 | -0.1615 | 0.155 | 0.0188 | **0.1838*** | **0.0135** | **3** | **0.0188** |
| 30s left lateral leg swings | -0.0493 | 0.6243 | 0.0438 | -0.1582 | 0.1637 | 0.0250 | -0.1006 | 0.1790 | 4 | 0.0250 |
| 30s right lateral leg swings | -0.0620 | 0.5377 | 0.0375 | -0.1042 | 0.3608 | 0.0375 | -0.0769 | 0.3047 | 5 | 0.0313 |
| Back strength assessment (kg) | -0.0996 | 0.3217 | 0.0313 | 0.1011 | 0.3755 | 0.0438 | -0.0134 | 0.8578 | 6 | 0.0375 |
| 30s resistance band pullbacks | -0.1378 | 0.1692 | 0.0250 | 0.1370 | 0.2285 | 0.0313 | -0.0101 | 0.8922 | 7 | 0.0438 |
| Kneeling plank (s) | 0.0219 | 0.8281 | 0.0500 | 0.0162 | 0.8872 | 0.0500 | -0.001 | 0.8942 | 8 | 0.0500 |

The second solution is to perform correlation analysis between low back pain and all physical fitness tests without FDR adjustment permitting certain risk of type 1 errors while decreasing potential risk of type 2 errors simultaneously.

Considering that our current study is preliminary and exploratory research, and there are few studies on pregnant women's low back pain and physical health, one of the purposes of our testing system is to serve as an early warning of pregnant women's physical level and symptoms during pregnancy, we are inclined to choose the second solution, which will bear certain risks of type 1 errors, but minimize the risk of type 2 errors. More importantly, it shows the integrity of our test system.

Of course with more data colletion and more exploration in test correlations and experimental designs, we will further improve the test accurency of our testing system.

3.Table 4 results - could the differences be explained by the extra weight of the baby later in pregnancy? For example the sit and reach test would be inhibited by the size of the bump. The difference in the kneel plank could also be due to the heavy baby. The balance tests appear to be inconclusive (differences for left leg for blind balance and not for right leg, differences for right-bird dog but not left) - again this could be due to running so many statistical tests - these difference might not be strong enough to be interest after adjusting for multiple testing.

**Answer:** yes, they could. Physical fitness is dynamic during pregnancy. The changes in physical fitness could be resulted from the changes of body shape and weight of pregnant women, and also because of fetal weight and liftstyle change.

Nowadays exercise during pregnancy has been a common sense but researches about scientific exercise are still in demand including comphrehensive physical fitness evaluation and scientific and effective exercise prescription. Even through some physical fitness evaluation methods have been published, we are trying to develop an objective comprehensive and practical evaluation system to enrich data about physical fitness of pregnant women and provide a foundation for follow-up studies on scientific exercise for pregnant women,

Here we reported results from only 180 participants. We believe that the change of physical fitness of pregnant women will be better explained with more data colletion and improvement of our comprehensive evaluation system in the near future.

4. Correlation Analysis: LBP is measured on an ordinal scale from 0 = no pain, 1 = very mild pain, 2 = moderate pain therefore a Pearson correlation or (Pearson partial correlation if looking to adjust for gestational week) is not appropriate.

An analysis suitable for ordinal data should be undertaken. Even if the Pearson analysis was appropriate, the results do not support the conclusions given in the abstract of strong negative correlations. The strongest correlation is -0.216 for 'right standing kick backs' which would considered a "weak" statistically significant correlation, a moderate/strong correlation would imply a Pearson correlation of at least 0.5 in magnitude.

**Answer:** following the reviewer’s suggestion, we reanalyzed the data with Spearman correlation tests. Table 5 has been updated in the manuscript.

We also accept the reviewer’s opinion about weak statistical significant correlation. We have modified the description of correlation result in the manuscript.

5. Conclusions drawn from the results: The correlation results are incorrectly reported as "strongly negatively correlated" - this needs to be much more considered after an appropriate analysis of the ordinal pain scale used.

**Answer**: we agree with the reviewer. Our conclusion has been modified. Thanks!

I would strongly recommend the authors work with a biostatistician to ensure the statistical analyses are correctly undertaken with careful considerations of the distribution of the data, outliers, the ordinal nature of the the pain scale and multiple testing as well as ensuring that any conclusions are not overstated.

**Reviewer #2:** Thank you for the opportunity to review the manuscript “The correlation between pregnancy-related low back pain and physical fitness evaluated by an index system of maternal physical fitness test”. It focuses on an important topic related to a majority of pregnant women. In my opinion the manuscript represents a good research practise. Nevertheless, following major consideration should be addressed:

1. Table 1: please, describe in more detail how the physical fitness tests were chosen into the testing battery. As described in previously, there is a wide variety of fitness testing protocols for pregnant women (see Romero-Gallardo L, Roldan Reoyo O, Castro-Piñero J, May LE, Ocón-Hernández O, Mottola MF, Aparicio VA, Soriano-Maldonado A. Assessment of physical fitness during pregnancy: validity and reliability of fitness tests, and relationship with maternal and neonatal health - a systematic review. BMJ Open Sport Exerc Med. 2022 Sep 23;8(3):e001318. doi: 10.1136/bmjsem-2022-001318. PMID: 36172399; PMCID: PMC9511659.).

**Answer:** thank you very much for recommending this paper that has been cited in our manuscript. We sorted out the current physical fitness tests for pregnant women, mainly focusing on contraindications for exercise during pregnancy, combining or revising some existing test systems, and paying less attention to factors such as the symptoms of lack of exercise, exercise demand, and test load during pregnancy. We believe that the goal of physical fitness testing during pregnancy is not only to understand physical fitness level, but also to set relevant standards through physical fitness level, to warn of adverse pregnancy symptoms caused by the decline of physical fitness level during pregnancy or possible adverse birth outcomes, so as to improve adverse pregnancy symptoms, optimize birth outcomes, alleviate birth fears, and enhance pregnancy experience.

Based on this, we tried to construct a testing system for pregnant women's physical fitness. The preliminary screening of pregnant women's physical fitness tests focused on the movements in multi-dimensions and multiple movement modes, in order to comprehensively understand the physical fitness level of pregnant women during pregnancy according to the physiological characteristics of pregnant women, exercise guidelines, exercise contraindications, exercise needs, existing physical fitness test indexes and other literature data as well as the interview opinions of experts related to pregnancy and exercise,

According to the above criteria, we preliminarily screened 47 tests. For example, we included 2-minute marching in place that is a common test for elderly people because it is practical for pregnant women and its intensity also meet the physical requirements and energy consumption during childbirth; 20-second hand grips and 20-second heel raises were designed to increase blood circulation in the hands and feet through quick movements of the hands and feet, improve the flexibility of the wrist and ankle joints, in order to alleviate the symptoms of hand and foot swelling during pregnancy.

Then we finalized with 23 tests after 3 rounds of Delphi survey by 10 experts (7 maternity medical specialists and 3 exercise experts). Using these 23 tests, we designed the standard testing process after consulting pregnant women's health and medical personnel and experts as well as pregnant individuals in terms of comfort and convenience of this testing battery.

Finally, we run it in practice. Over 300 pregnant individuals have completed this set of tests by now. It turns out that the practical operation runs smoothly. All the pregnant women complete the tests and have no discomfort during testing. Additionally, there is no significant difference in fetal heart rate before and after testing.

2. Can be the results of sit-and-reach test affected by the size of the pregnant belly or it reflects the change in flexibility of pregnant women?

**Answer:** yes, the sit-and-reach test is to evaluate body’s flexibility of pregnant women at current gestational week on testing day indicating their movement range and living convenience level.

Certainly, the result of sit-and-reach test is dramatically effected by gestational week. We attempt to roughly differentiate between middle gestation and late gestation in this study. With increasing data collection, we will gradually narrow gaps between groups and complete more accurate studies by month and gestation week.

3. What is the validity and reliability of the physical fitness test battery used in the manuscript?

**Answer:** regarding the validity of the physical fitness tests, as we mentioned in question 1. Firstly, we screened 47 preliminary tests according to relevant criteria. Secondly, 23 physical tests were finalized through 3 rounds of Delphi survey conducted by 10 multidisciplinary experts (7 maternity medical specialists and 3 exercise experts). Finally, operation of physical fitness testing system was verified through physical fitness evaluation on pregnant women and testing feedback from the pregnant women participants. In summary, we mainly ensure the validity of physical indexes through the screening of pregnancy-related tests, multidisciplinary expert demonstration and practical operation effects.

Regarding the reliability of the physical fitness tests, firstly we choose multiple representative tests during the preliminary screening; secondly, some tests with low reliability were kicked out after the repetitive test during further screening of non-representative tests; thirdly, all selected tests are organized and performed with a computational system. For example, before the test, each participant is informed of the method used and watch a video showing the standard movement to ensure the consistency of testing movement understanding; during the test, the pre-established computational system unifies the testing process for every participant with the same total testing time and same interval time to make sure the consistency of the test process.

We have published the screening and construction of the physical fitness evaluation for pregnant women in Chinese Journal of Perinatal Medicine (DOI: 10.3760 / cma. J.c. n113903-20210412-00338). Only abstract but not full article is in English.

Although our physical fitness test system constructed is running well at this stage, we know that, with more test data collected and deepening research on the correlation of tests and experimental studies, we will further improve the representativeness and feasibility of tests and constantly increase the reliability and validity of the entire physical fitness test system.

4. Table 3: as low back pain intensity 4 and 5 were one of the exclusion criteria, do not display them at the table. Information about the number of participants prepared to participate in the study and those who were excluded because the high pain intensity would bring an additional valuable information.

**Answer:** we have removed the display about low back pain intensity 4 and 5 in the manuscript. In ethical perspective, the pregnant women who have severe pain are not allowed to take risks participanting our evaluation system, so we appreciate the reviewer’s suggestion and will collect this information in the following experimental execution.

As non-native English speaker, I found some language mistakes. Consider language editing of the final manuscript.

**Answer:** we have went through the manuscript and have corrected mistakes we found. Finally, thank you for your time and valuable comments.
